# Transductive Transfer Learning for Domain Adaptation in Brain Magnetic Resonance Image Segmentation

**DOI:** 10.3389/fnins.2021.608808

**Published:** 2021-04-29

**Authors:** Kaisar Kushibar, Mostafa Salem, Sergi Valverde, Àlex Rovira, Joaquim Salvi, Arnau Oliver, Xavier Lladó

**Affiliations:** ^1^Institute of Computer Vision and Robotics, University of Girona, Girona, Spain; ^2^Computer Science Department, Faculty of Computers and Information, Assiut University, Asyut, Egypt; ^3^Magnetic Resonance Unit, Department of Radiology, Vall d'Hebron University Hospital, Barcelona, Spain

**Keywords:** deep learning, domain adaptation, magnetic resonance imaging, brain, segmentation, sub-cortical structures, white matter hyperintensities, transductive learning

## Abstract

Segmentation of brain images from Magnetic Resonance Images (MRI) is an indispensable step in clinical practice. Morphological changes of sub-cortical brain structures and quantification of brain lesions are considered biomarkers of neurological and neurodegenerative disorders and used for diagnosis, treatment planning, and monitoring disease progression. In recent years, deep learning methods showed an outstanding performance in medical image segmentation. However, these methods suffer from generalisability problem due to inter-centre and inter-scanner variabilities of the MRI images. The main objective of the study is to develop an automated deep learning segmentation approach that is accurate and robust to the variabilities in scanner and acquisition protocols. In this paper, we propose a transductive transfer learning approach for domain adaptation to reduce the domain-shift effect in brain MRI segmentation. The transductive scenario assumes that there are sets of images from two different domains: (1) source—images with manually annotated labels; and (2) target—images without expert annotations. Then, the network is jointly optimised integrating both source and target images into the transductive training process to segment the regions of interest and to minimise the domain-shift effect. We proposed to use a histogram loss in the feature level to carry out the latter optimisation problem. In order to demonstrate the benefit of the proposed approach, the method has been tested in two different brain MRI image segmentation problems using multi-centre and multi-scanner databases for: (1) sub-cortical brain structure segmentation; and (2) white matter hyperintensities segmentation. The experiments showed that the segmentation performance of a pre-trained model could be significantly improved by up to 10%. For the first segmentation problem it was possible to achieve a maximum improvement from 0.680 to 0.799 in average Dice Similarity Coefficient (DSC) metric and for the second problem the average DSC improved from 0.504 to 0.602. Moreover, the improvements after domain adaptation were on par or showed better performance compared to the commonly used traditional unsupervised segmentation methods (FIRST and LST), also achieving faster execution time. Taking this into account, this work presents one more step toward the practical implementation of deep learning algorithms into the clinical routine.

## 1. Introduction

Medical image segmentation is a pivotal task in diagnosis, treatment, and surgical planning, and monitoring disease progression over time. Quantification of brain structures and brain lesions from Magnetic Resonance Images (MRI) is crucial as they are biomarkers for neurological and neurodegenerative disorders. However, manually annotating MRI images is a time-consuming and a laborious task, which has to be done by experts with knowledge in disease-specific aspects and anatomy. Therefore, there is a need for accurate and automated methods to carry out different segmentation problems in brain MRI—e.g., brain structure (González-Villà et al., 2016), multiple sclerosis (MS) (Garćıa-Lorenzo et al., 2013), and brain tumour (Bakas et al., 2018).

In recent years, deep learning methods—in particular, Convolutional Neural Networks (CNNs)—have shown a remarkable advance in the field of brain MRI segmentation for many different applications (Akkus et al., 2017; Bernal et al., 2019). Unlike the traditional hand-crafted features, CNNs learn task-specific features directly from observed data (LeCun et al., 2015). Most CNN based approaches for medical image segmentation in literature are usually trained and tested with images that share common characteristics—the same scanner and acquisition protocol. However, the performance of such pre-trained networks decline when tested on images with different MRI characteristics, i.e., images from a different domain (MRI scanner, protocol). Deep learning methods cannot generalise to unseen domains where the image scans vary in brightness, contrast, and resolution. Therefore, the network has to be re-trained using the images from this new domain, requiring expert annotated labels. This commonly faced issue is known as the domain-shift problem, which hinders the applicability of deep learning methods in practice. Moreover, the data-driven nature, which demands a vast amount of expert annotated images, often makes fully retraining a CNN impossible.

Transfer learning strategy is an effective way to adapt a pre-trained neural network to a new domain. This procedure consists in retraining only a few last layers, which can be done using a remarkably smaller number of annotated images (Ghafoorian et al., 2017; Valverde et al., 2019). However, it is not always possible to obtain even a few images to perform transfer learning for domain adaptation. Therefore, other unsupervised domain adaptation methods are active research topics in medical image analysis. A recent work of Orbes-Arteainst et al. (2019) proposed an unsupervised domain adaptation approach in a similar fashion to transfer learning with teacher-student learning strategy. The authors used knowledge-distillation technique where a supervised teacher model is used to train a student network by generating soft labels for the target domain.

In general, unsupervised domain adaptation methods could be categorised into: (1) image-level, where the images of two domains are harmonised to share similar characteristics; and (2) feature-level approaches where the CNN itself is adapted to be more invariant to different imaging domains. Common approaches for the image-level domain adaptation include traditional pre-processing steps (Shah et al., 2011; Fortin et al., 2016). One of the common challenges of the traditional approaches include image artefacts that may appear during intensity transformations that reduce the image quality. Moreover, it was shown (Kushibar et al., 2019) that approaches such as standardising images using the Nyúl histogram matching (Nyúl et al., 2000) or mixing datasets from different domains during training cannot overcome the effect of the domain-shift.

More complex Generative Adversarial Networks (GAN) (Goodfellow et al., 2014) based approaches have also been introduced for translating images into a new target domain. However, most of the works in the literature propose synthesising images from a different imaging modality. For example, Huo et al. (2018), utilise CycleGAN framework to generate CT images from MRI to allow splenomegaly segmentation without using manual annotation on CT. Also, Zhang et al. (2018) proposed a modified CycleGAN approach for multi-organ segmentation on X-ray images using Digitally Reconstructed Radiographs by performing pixel-to-pixel style transfer from one modality to another. Although such approaches have shown promising results, there is still a lack of GAN based methods for single-modality image harmonisation.

Some feature-level domain adaptation methods have also been proposed in recent years. Such methods employ a transductive learning strategy for domain adaptation. In the transductive scenario, the images without expert annotations from unseen domain are included in the training process with the aim to minimise the domain-shift effect. Adversarial training of the network is a well-known transductive learning method. Similarly to GAN architectures, the training strategy consists of two network paths: one for classifying the input patch, and another to force the network to learn domain-invariant features by discriminating source and target domains. Recent work of Kamnitsas et al. (2017) utilises an adversarial training approach for unsupervised domain adaptation from Gradient Echo images to Susceptibility Weighted Images for brain lesion segmentation task. Moreover, an adversarial domain adaptation from Whole Slide pathology to Microscopy images has been studied in Zhang et al. (2019). Chen et al. (2020) proposed simultaneous image to image translation and domain alignment between CT and MRI images using a modification of a CycleGAN for cardiac and abdominal multi-organ segmentation. However, more investigation is needed for the adversarial training for domain adaptation for a scenario where the domain difference is subtle—i.e., multi-site and single-modality images.

There are some drawbacks of GAN based and adversarial training strategies. These methods are usually formulated as a competition between two agents: discriminator and segmenter (Yi et al., 2019). In general, the objective for the latter can vary according to the task (e.g., it is called generator for image synthesis), but in most cases the objective of the former is to differentiate between two distributions. In this non-convex min-max formulation, the training of the network can be difficult and unstable, which requires a careful selection of architecture, weight initialisation, and hyper-parameter tuning (Roth et al., 2017). For example, Li et al. (2020) proposed an adversarial approach for single modality domain adaptation with flip-label technique where the labels of the discriminator model were partly inverted during training to minimise over-fitting.

Other feature-level transductive domain adaptation methods perform domain distribution discrepancy minimisation to learn domain-invariant features. Most of the advancements of such approaches are done for computer vision with natural images (Damodaran et al., 2018; Rozantsev et al., 2018; Kang et al., 2019). However, only a few works have been proposed in medical imaging field for single-modality images. One of the recent domain adaptation approaches is the work of Ackaouy et al. (2020) for multi-site brain multiple sclerosis lesion segmentation. The authors adopted a joint distribution optimal transport framework proposed in Damodaran et al. (2018) to compare the source and target distributions and bring them closer in a feature-level.

In this paper, we propose a feature-level transductive domain adaptation method that can be trained without extensive hyper-parameter tuning. Similarly to Ackaouy et al. (2020), our proposed method aligns the network feature distributions between two different domains by forcing the convolutional and fully connected layers to produce similar activation maps by minimising the histogram distribution differences. The images from a new domain are incorporated within training transductively and do not require expert annotated ground truths. To show its robustness and applicability, we utilise and evaluate our domain adaptation approach for two active brain MRI segmentation problems—brain sub-cortical structure segmentation and brain White Matter Hyperintensities (WMH) segmentation. We compare the performance of our proposal with segmentation results without domain adaptation as well as the unsupervised state-of-the-art approaches for each problem: (1) FIRST (Patenaude et al., 2011) for sub-cortical structure segmentation; and (2) LST (Schmidt and Wink, 2019) for WMH lesion segmentation.

## 2. Datasets and Pre-Processing

We used publicly available and in-house datasets to test the performance of our proposed method for the selected segmentation tasks. Internet Brain Segmentation Repository^1^ (IBSR) and Multi-Atlas Labelling Challenge (MICCAI2012) datasets (Landman and Warfield, 2012) were used for the sub-cortical structure segmentation problem. For the WMH segmentation, one dataset comes from an international WMH lesion segmentation challenge (Kuijf et al., 2019), and another from the Vall d'Hebron Hospital Centre (Barcelona, Spain). More information for each dataset is given below.

### 2.1. Sub-cortical Brain Structure Segmentation

#### 2.1.1. Motivation

The sub-cortical structures are located beneath the cerebral cortex and include the thalamus, caudate, putamen, pallidum, hippocampus, amygdala, and accumbens. Their deviations in volume over time are considered as biomarkers of neurological diseases such as bipolar disorder (Frazier et al., 2005), Alzheimer's (De Jong et al., 2008), schizophrenia (Rimol et al., 2010), Parkinson's disease (Mak et al., 2014), multiple sclerosis (Houtchens et al., 2007), and are used for pre-operative evaluation and surgical planning (Kikinis et al., 1996), and longitudinal monitoring for disease progression or remission (Storelli et al., 2018). The volumes of the sub-cortical structures differ drastically, in average, 8,500 and ≈550*mm*^3^ for largest thalamus and smallest accumbens structures, respectively. This makes the segmentation task more challenging by introducing an unbalanced class problem.

#### 2.1.2. Multi-Atlas Labelling Challenge—MICCAI 2012

The MICCAI 2012 dataset consists of 35 T1-w images in total with 15 training and 20 testing MRI scans. In our experiments, we used the 20 testing set only for testing purposes and they were not included in the training or validation processes in order to follow the rules of the Multi-Atlas Labelling challenge. All T1-w scans have 1*mm*^3^ isotropic resolution and image dimensions are 256 × 256 × 256 voxels. All images in this dataset were acquired using the same Siemens (1.5 T) MRI scanner. Manually annotated ground truth masks were provided for 134 structures in total, from which 14 classes were extracted for the seven sub-cortical structures corresponding to the left and right hemispheres.

#### 2.1.3. Internet Brain Segmentation Repository—IBSR

The IBSR dataset contains 18 T1-w images in total which are publicly available under the Creative Commons: Attribute license (CC-BY, 2020) as part of the Child and Adolescent Neuro-Development Initiative (CANDI) (Kennedy et al., 2012). The image volumes in this dataset come in three different resolutions—0.84 × 0.84 × 1.5, 0.94 × 0.94 × 1.5, and 1 × 1 × 1.5*mm*^3^—and were acquired using two different MRI scanners—GE (1.5 T) and Siemens (1.5 T). Manual annotations for all IBSR images were provided by the Center for Morphometric Analysis at Massachusetts General Hospital and consist of 43 different structures in total (Rohlfing, 2012). For our experiments, we selected the 14 labels corresponding to seven sub-cortical structures with left and right parts separately.

### 2.2. White Matter Hyperintensity Lesion Segmentation

#### 2.2.1. Motivation

White Matter Hyperintensities are brain lesions that appear bright in T2-weighted and Fluid Attenuated Inversion Recovery (FLAIR) sequences. The presence of the WMH lesions can be from different factors including small vessel disease (Van Norden et al., 2011), multiple sclerosis (Kutzelnigg et al., 2005), stroke or dementia (Debette and Markus, 2010). Monitoring the lesion load and appearance of new lesions is important for diagnosis, longitudinal analysis, and treatment planning (Polman et al., 2011). In contrast to the sub-cortical structure segmentation task, WMH lesions can appear anywhere in the brain within the white matter and can be of different shape and size. Taking into account the importance of lesion load quantification as biomarkers for different neurodegenerative disorders, this task is a relevant and a challenging segmentation problem.

#### 2.2.2. White Matter Hyperintensities Segmentation Challenge—WMH 2017

The WMH 2017 dataset provides T1-w and FLAIR scans for 60 patients in total and were acquired from three different sites^2^: (1) UMC Utrecht—3T Philips Achieva with 1*mm*^3^ isotropic T1-w and 0.96 × 0.95 × 3.0*mm*^3^ resolution FLAIR sequences; (2) NUHS Singapore—3 T Siemens TrioTim with 1*mm*^3^ isotropic T1-w and 1.0 × 1.0 × 3.0*mm*^3^ resolution FLAIR sequences; and (3) VU Amsterdam—3 T GE Signa HDxt with 0.94 × 0.94 × 1.0*mm*^3^ T1-w and 0.98 × 0.98 × 1.2*mm*^3^ resolution FLAIR sequences. All T1-w volumes were re-sampled to their corresponding FLAIR images. Ground truth labels for the WMH lesions were manually annotated and peer-reviewed by experts (Kuijf et al., 2019).

#### 2.2.3. In-House Dataset—Vall d'Hebron Hospital, Barcelona (VH)

This dataset contains MRI images for 28 patients with clinically isolated syndrome or early relapsing multiple sclerosis. All MRI scans were acquired in the same 3T Siemens TrioTim scanner that include T1-w and FLAIR images with 1.0 × 1.0 × 1.2 and 0.49 × 0.49 × 3.0*mm*^3^ resolutions, respectively. Similarly to the WMH 2017 dataset, all T1-w images were re-sampled to their corresponding FLAIR sequences. The WMH lesions were manually annotated and peer-reviewed by experts from the Vall d'Hebron Hospital centre. The MRI volumes were included in this dataset after the patients gave their informed consent which was approved by the Institutional Review Board.

## 3. Methods

### 3.1. CNN Architecture

**Figure 1:**
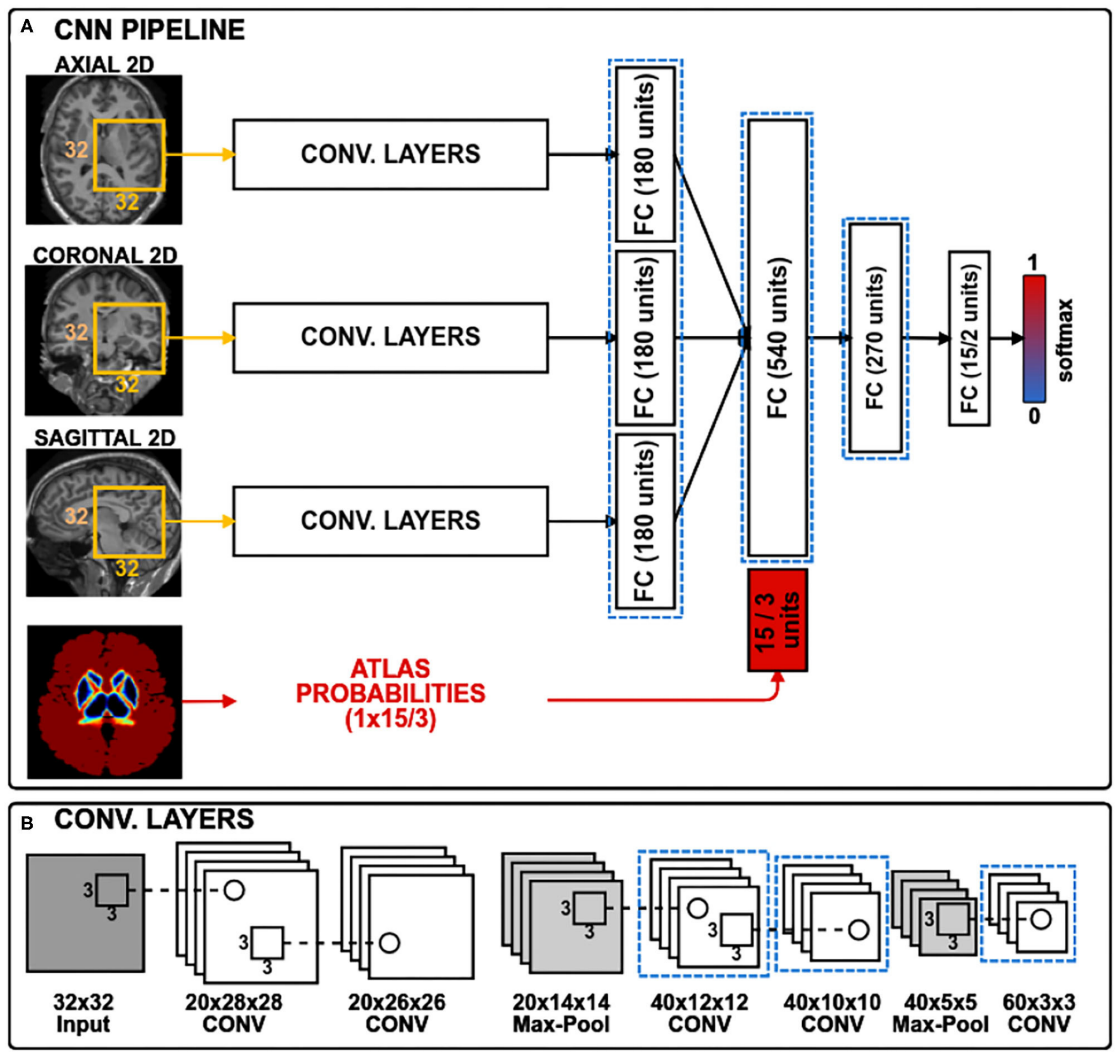
The CNN architecture has three convolutional branches and a branch for spatial priors. 2D patches of size 32 × 32 pixels are extracted from three orthogonal views of a 3D volume. For sub-cortical structure segmentation, the spatial prior branch accepts a vector of size 15 with atlas probabilities for each of the 14 structures plus the background, whereas for the WMH lesion segmentation the vector size is three corresponding to white matter, gray matter and cerebrospinal fluid. Histogram loss is computed from the activation maps of the layers, highlighted with dashed blue rectangles. **(A)** CNN pipeline; **(B)** Convolutional layers.

In this work, to study the domain-shift problem and to evaluate our transductive domain adaptation approach, we took the recent architecture proposed in Kushibar et al. (2018), which achieved state-of-the-art performance for sub-cortical brain structure segmentation. The CNN is shown in Figure 1 and consists of three paths to process 2D patches of size 32 × 32. Each path is equipped with five convolution layers, which are followed by a fully connected layer. The outputs of these paths are concatenated together with an additional 15 units corresponding to atlas probabilities for the 14 sub-cortical brain structures and the background. According to Kushibar et al. (2018), incorporation of the atlas probabilities as spatial prior to guide the network significantly improved the performance. For the case of WMH lesion segmentation the number of units for the atlas probabilities is changed to three, which correspond to white matter, grey matter, and cerebro-spinal fluid probabilities. Finally, it is followed by fully connected layers to mine and classify the produced output from the preceding layers. Three 2D patches are extracted for every voxel from the axial, sagittal and coronal views of a 3D volume, making 2.5D patch samples. Next, each orthogonal 2D patch of the 2.5D sample is inputted to the three paths of the CNN. Although full 3D patches contain more surrounding information per voxel, it is more memory-demanding than using 2D patches in voxel-wise segmentation setup. Therefore, employing 2.5D patches is a good trade-off between memory and contextual information for the network (Kushibar et al., 2018).

### 3.2. Pre-processing

Some commonly used image pre-processing techniques were applied to all of the images in the four datasets. First of all, we non-linearly registered atlas probabilities to the images using the fast free-form deformation method (Modat et al., 2010) that was implemented by the NiftyReg tool^3^. We used the well-known Harvard-Oxford probabilistic atlas (Caviness Jr et al., 1996) distributed with the FSL (v5.0) tool^4^. Note that the number of probabilistic maps for the structure segmentation problem is 14, whereas it is 3 for the WMH lesion segmentation which correspond to the three tissue types. In the next step, we skull-stripped all the MRI volumes—i.e., removed non-brain structures, such as the eyes and skull—using the ROBEX (v1.2) tool (Iglesias et al., 2011). Additionally, we performed bias-field correction to remove intensity inhomogeneities from the images using the FSL-FAST tool. All subject volume intensities were normalised to have a zero mean and unit variance before training and testing the pipeline. Note that the images provided in WMH 2017 Challenge were already bias-field-corrected, co-registered, and the 3D T1-weighted images were aligned (re-sampled) with the FLAIR images by the organisers (Kuijf et al., 2019).

### 3.3. Initial Training

Before adapting the network to a new domain for a certain task, we assume that the network is pre-trained for the same segmentation problem. Therefore, in this section, we describe how the initial training was done for each segmentation task.

For the sub-cortical structure segmentation problem, we used the same initial training process as described in Kushibar et al. (2018). All samples were extracted from the 14 sub-cortical structures, and the background (negative) samples were selected only from the structure boundaries within a five-voxel margin. Extracting the negative samples in this way allows the network to learn the most difficult areas of the region of interest that correspond to the structure borders. Next, the atlas probabilities for 14 structures and the background are extracted, corresponding to all training samples and making a vector of size 15. These probabilities provide the network with spatial information and guide it to overcome intensity-based difficulties in some MRI volumes such as imaging artefacts and abnormalities caused by neurological diseases as black holes that appear next to the structures (Kushibar et al., 2018).

For the WMH lesion segmentation task, we used a cascaded training strategy as described in Valverde et al. (2017), where the network was trained in two stages. In the first step, the network is trained with a balanced number of samples extracted from all lesion voxels and an equal number of negative voxels randomly selected from non-lesion parts of the brain. Then, the same set of training images is segmented to obtain initial lesion masks. In the second stage, the network is also trained with a balanced set containing all lesion samples, however, the negative samples are extracted only from the voxels that were incorrectly classified in the first segmentation stage. This step is equivalent to a false positive reduction step.

For both tasks, the training samples were extracted along with their atlas probabilities, and randomly split into training and validation sets with 75 and 25% proportions, respectively. The training of the network was performed in batches of 128 for 200 epochs. An early-stopping protocol was defined with patience 20—i.e., the training stops if no increase was observed in the validation accuracy for 20 consecutive epochs. Optimisation was conducted for the categorical cross-entropy loss function using the Adam optimisation method (Kingma and Ba, 2014) with a learning rate of 10^−2^.

### 3.4. Transductive Domain Adaptation

**Figure 2:**
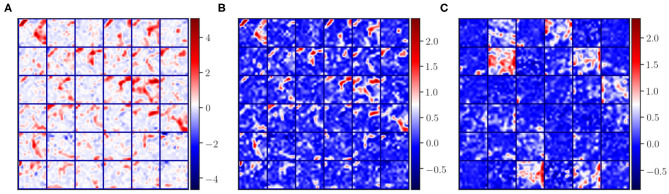
Illustration of some activation maps for **(A)** source, **(B)** source after applying histogram matching to the target, and **(C)** target. Here, 36 example activation maps from the third convolutional layer are shown with the “*seismic”* color-map to visually emphasise the differences in magnitudes of the activation maps.

In the problem of domain adaptation we refer to source and target domains, where the former is the image domain with ground truth labels used in the initial training phase and the latter represents the new image domain without ground truth masks. When looking at the activation maps of the convolutional layers extracted for source and target, we can observe the differences in intensity distributions as shown in Figure 2. As can be seen in Figure 2A, the magnitude of the activation maps for the source appear brighter compared to the target (Figure 2C). This demonstrates how the domain-shift problem affects the CNN in the feature level. Thus, the fully connected layers, which are used to mine these extracted features, cannot generalise to a different domain. When performing traditional transfer learning by re-training the last few layers of the network, we are adapting the fully connected part to better interpret the changes shown in Figures 2A,C. However, ground truth labels are not always available to perform such transfer learning for domain adaptation.

**Figure 3:**
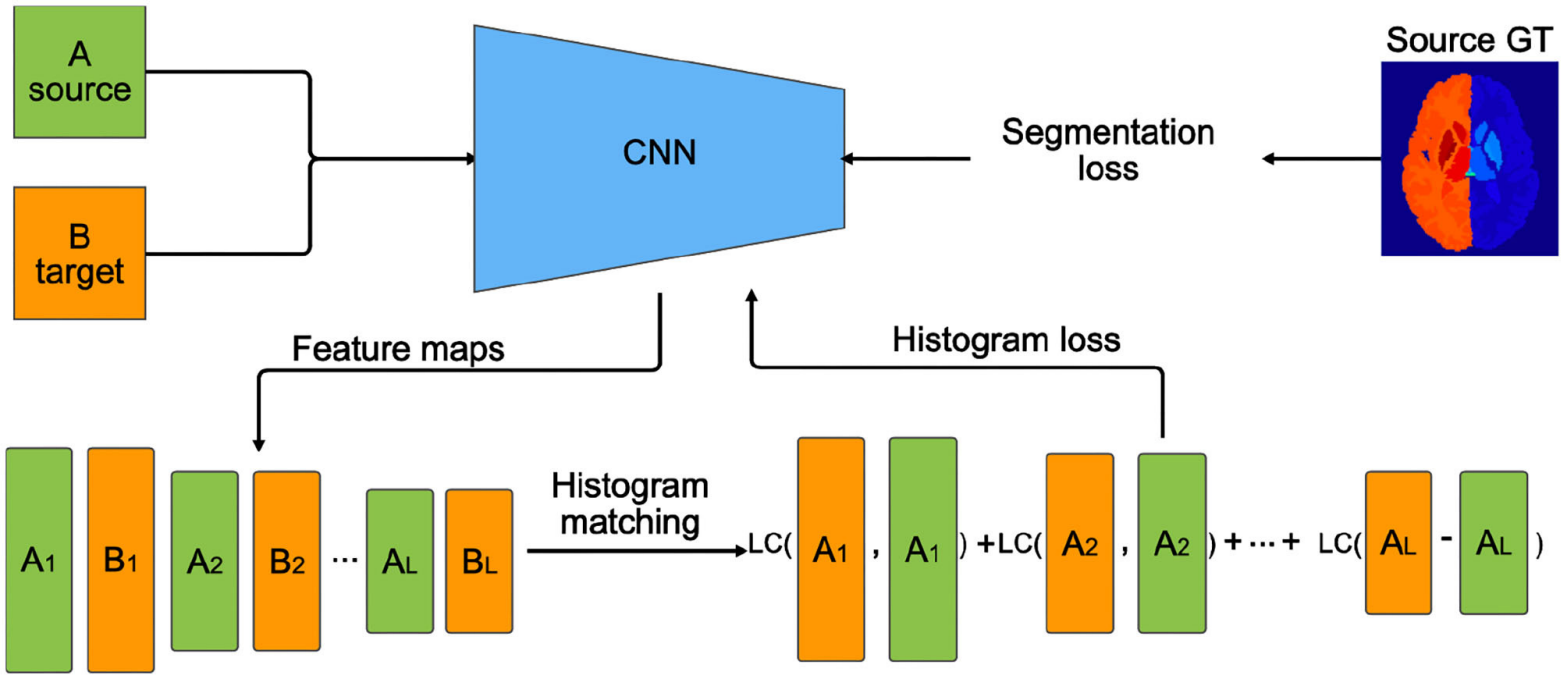
Transductive domain adaptation training pipeline using histogram loss. *A*_*i*_ and *B*_*i*_ are feature maps extracted from the *ith* layers of the CNN. *L* is the number of layers on which the histogram loss is computed. Segmentation loss, in our case cross-entropy loss, is computed using the source ground truth (GT) labels. LC (LogCosh)—logarithm of hyperbolic cosine function.

In this paper, we propose an alternative approach to traditional transfer learning by adapting the feature maps in the network instead of retraining the last few layers. Figure 3 illustrates the transductive training process pipeline. First, features maps are extracted from several layers of the CNN for source and target training images. Then, the activation maps from the source domain are mapped to the features of target domain using a histogram matching technique. Next, we calculate the distance from the original source features to the histogram matched feature distributions. This difference is back-propagated as a histogram loss to encourage the network to produce feature maps similar to the target.

Let *L*_*i*_ be the layers of the CNN that we want to apply the histogram loss, and let us define *A*_*i*_ and *B*_*i*_ as the activation maps from the source and target samples for the *ith* layer, respectively. Then, the histogram loss is computed as:

(1) $$\begin{array}{r} L_{hist}=\overset{L}{\underset{i}{\sum }}LogCosh\left(A_{i},H\left(A_{i},B_{i}\right)\right), \end{array}$$

where, *H*(·, ·) is a function that applies a regular histogram mapping from source *A*_*i*_ to *B*_*i*_ target, and *LogCosh* is a logarithm of hyperbolic cosine that mostly works like the mean squared error but less affected by occasional large differences in the feature maps. In this form, the histogram loss is differentiable, and the loss can be computed easily by storing the histogram matched matrices for *A*_*i*_ in memory. Moreover, with this approach, the images from the target domain are included in training in a transductive manner in the feature level with no requirement for ground truth labels. An example of histogram matched feature maps of the source samples is shown in Figure 2B. Here, we can observe that the spatial integrity is the same as the original features (Figure 2A) and the intensity distribution is similar to the target features (Figure 2C).

Note that overall, we aim to minimise the following loss function:

(2) $$\begin{array}{r} L_{total}=L_{ce}+\lambda L_{hist}, \end{array}$$

where $L_{ce}$ is a cross-entropy loss and λ is a hyper-parameter to weight the effect of the histogram loss. The cross-entropy loss is computed using the source images with ground truth labels. Inclusion of this term is important to make the network learn to adapt to the changes in the feature maps after the histogram loss takes effect.

In our experiments, setting λ to be 1.0 showed the best results. Also, it has to be noted that the performance of the method was not very sensitive to the values within 1±0.6. However, much larger or smaller values caused overshooting or diminished the effect of histogram loss during training. One could increase or decrease this weight out of the suggested range when applying for a different task that was not addressed in this study to change the influence of the histogram loss. The learning rate was reduced to 10^−4^ to avoid rapid weight updates. Applying histogram matching per sample could be limited due to the variance of histograms from different locations in the brain. Therefore, the histogram loss is computed over a batch—in our case batches of 32—hence, the loss is computed over a distribution rather than per sample, which we note as a necessary requirement. We empirically chose the last three convolutional, and all fully connected layers except for the last classification layer to compute the histogram loss as shown in Figure 1 with dashed blue rectangles. For both segmentation tasks, using only one image from source and target sets was sufficient to perform the domain adaptation.

### 3.5. Network Testing

To perform a segmentation with a trained model, all 2.5D patches and corresponding atlas probabilities are extracted from an MRI volume, then passed through the CNN to obtain a probability map for each patch.

For the sub-cortical structure segmentation, the final label is defined using the *argmax* function. For this task, we used patches only from a region of interest (ROI) defined by a mask from the dilated atlas probabilities of the structures. In doing so, we were able to speed up the segmentation process drastically because the sub-cortical structures are located in the central part of the brain. Since the network is well trained to classify the borders of the structures, there may appear some wrongly classified voxels, which are removed by keeping only the largest volume for each class.

For WMH lesion segmentation, we use all the available brain patches because lesions can be in any place in the brain within the white matter. The obtained output probability maps from the CNN are thresholded to produce binary outputs with lesion candidates. Then, all lesion candidates that are outside the white matter defined by the registered probabilistic atlas, as well as candidates that have a volume less than 3*mm*^3^ are removed (Filippi et al., 2016).

### 3.6. Experiments and Evaluation

In this section, we describe the experimental setups used to test our approach for the two different segmentation tasks.

For the sub-cortical structure segmentation problem, we set up two pre-trained baseline models with MICCAI 2012 and IBSR dataset images as source. Then, domain adaptation was carried out in three ways: (1) from IBSR baseline to MICCAI 2012; (2) from MICCAI 2012 baseline to IBSR-GE; and (3) from MICCAI 2012 baseline to IBSR-SIEMENS. We separated the IBSR dataset into the IBSR-GE and IBSR-SIEMENS sub-groups according to the scanner manufacturer. This division was done to perform evaluation using the images with inter-scanner variability.

For the WMH lesion segmentation task we defined two pre-trained baseline models with WMH 2017 and VH dataset images as source. Then, we applied domain adaptation in four ways: (1) from WMH 2017 model to VH; (2) from VH model to UMC Utrecht site; (3) from VH model to Singapore site; and (4) from VH model to VU Amsterdam site.

Performing the domain adaptation for this experimental setup ensures that the source and target domains are different, and offers a realistic application of our proposal. We also compare our results with well-known unsupervised segmentation methods for both tasks. For the sub-cortical structure segmentation, we used the FSL-FIRST with default parameters, whereas for WMH lesion we used the LST method with κ thresholds empirically set to 0.4 and 0.1, which showed the best segmentation result for VH and WMH 2017 datasets, respectively.

For the sub-cortical structure segmentation task we reported the Dice Similarity Coefficient (DSC), since it is the most commonly used metric in the literature. The DSC is an overlap measurement that shows how well the automated segmentation is aligned with the gold standard; zero being no overlap and 1.0 full overlap. For the WMH lesion segmentation, along with the overlap DSC measure, we also used the common metrics of detection—True Positive Rate (TPR) and False Positive Rate (FPR)—which indicate the method's performance for detection and correct classification of the lesion candidates. Both the TPR and FPR values range between zero and one, where higher values are better for TPR and lower is better for FPR. Also, we used the common F-score metric that incorporates both measures to show classifier accuracy in correctly detecting lesions, and it ranges from zero (low) to one (high).

We used the pairwise non-parametric Wilcoxon signed-rank test (two-sided) to compare the statistical significance of our results with respect to the results of the pre-trained baseline model without domain adaptation and the state-of-the-art tools. The results were considered significant for (*p* < 0.05). Moreover, we perform Bonferroni correction to the significance levels when comparing structure-wise and lesion-wise detection and segmentation for both of the selected tasks to counteract the multiple comparisons problem. Therefore, the differences will be assumed to be significant for (*p* < 0.0036) and (*p* < 0.0125) for sub-cortical structure and WMH lesion segmentation tasks, respectively.

All the experiments were run using a machine with a 3.40-GHz CPU clock and on a single TITAN-X GPU (NVIDIA corp, United States) with 12 GB of RAM memory. The network was implemented using the Keras (Chollet et al., 2018) deep learning library with Tensorflow backend^5^.

## 4. Results

### 4.1. Sub-cortical Structure Segmentation

**Figure 4:**
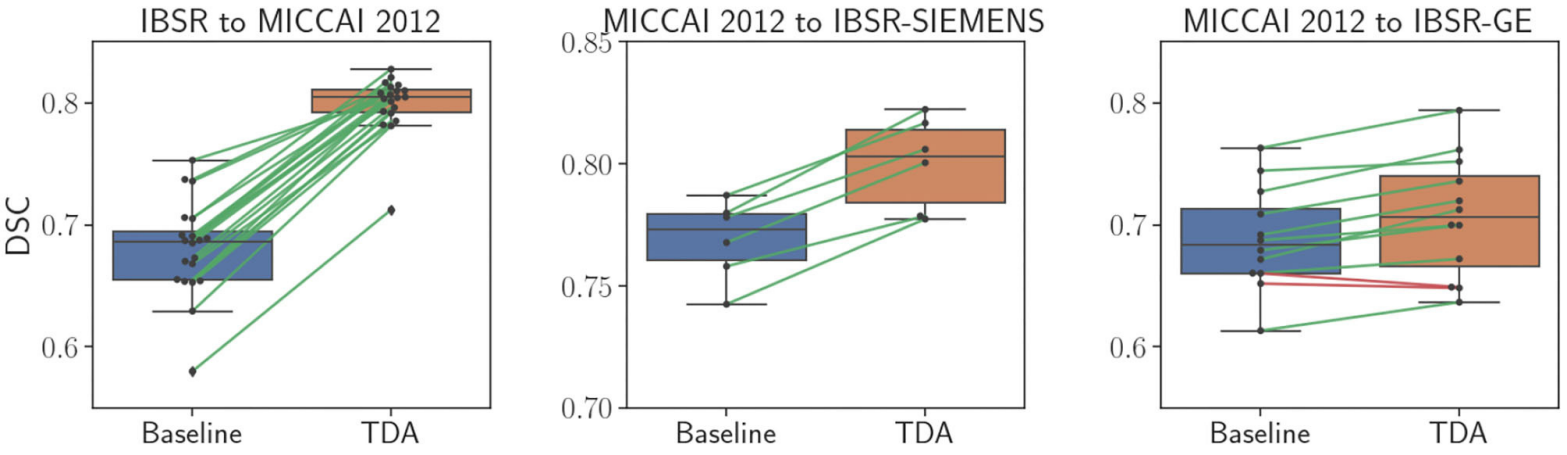
Comparison of sub-cortical structure segmentation between direct testing (Baseline) and after domain adaptation (TDA). Black dots refer to each subject volume in the target dataset. The connecting lines show correspondence for improved (green) and decreased (red) DSC values.

Table 1 shows the DSC results of the pre-trained baseline model without domain adaptation, proposed domain adaptation method, and FIRST for three datasets. Also, Figure 4 illustrates segmentation improvements from the baseline after applying domain adaptation with subject-wise correspondence of the volumes in the target dataset. When testing the method on the first set, where IBSR was source and MICCAI 2012 was target, significant improvement in the overall result was observed after applying the domain adaptation, reaching a DSC of 0.799 compared to the baseline segmentation with the DSC score of 0.680 (*p* = 2.8 × 10^−27^). The average DSC of our method was similar to FIRST and the difference was not statistically significant (*p* = 0.160). Significant structure-wise improvements in the results were also observed for most of the structures when domain adaptation was applied: left thalamus (*p* = 8.9 × 10^−5^), right thalamus (*p* = 8.9 × 10^−5^), left pallidum (*p* = 8.9 × 10^−5^), right pallidum (*p* = 8.9 × 10^−5^), left hippocampus (*p* = 1.9 × 10^−4^), right hippocampus (*p* = 0.002), and right amygdala (*p* = 8.9 × 10^−5^).

**Table 1:** DSC results with standard deviations for the pre-trained baseline model without domain adaptation, transductive domain adaptation (TDA), and unsupervised FIRST method for two-way validation: from IBSR to MICCAI 2012; from MICCAI 2012 to IBSR-SIEMENS; and from MICCAI 2012 to IBSR-GE.

	**IBSR to MICCAI 2012**			**MICCAI2012 to IBSR-SIEMENS**			**MICCAI 2012 to IBSR-GE**		
	**Baseline**	**TDA**	**FIRST**	**Baseline**	**TDA**	**FIRST**	**Baseline**	**TDA**	**FIRST**
Tha.L	0.301 ± 0.195	0.843 ± 0.028*	**0.889** **±** **0.017**	0.842 ± 0.029	0.873 ± 0.023	**0.892** **±** **0.022**	0.681 ± 0.102	0.699 ± 0.111*	**0.894** **±** **0.015**
Tha.R	0.085 ± 0.203	0.857 ± 0.022*	**0.890** **±** **0.018**	0.823 ± 0.026	0.886 ± 0.016	**0.889** **±** **0.014**	0.701 ± 0.108	0.736 ± 0.124*	**0.882** **±** **0.011**
Cau.L	**0.867** **±** **0.052**	0.861 ± 0.057	0.797 ± 0.117	0.862 ± 0.020	**0.887** **±** **0.014**	0.805 ± 0.028	0.801 ± 0.074	**0.836** **±** 0.046*	0.771 ± 0.047
Cau.R	**0.873** **±** **0.040**	0.865 ± 0.044	0.837 ± 0.046	0.860 ± 0.011	0.864 ± 0.015	**0.892** **±** **0.016**	0.828 ± 0.029	0.834 ± 0.025	**0.860** **±** **0.026**
Put.L	0.888 ± 0.023	**0.893** **±** **0.022**	0.860 ± 0.080	**0.891** **±** **0.024**	0.888 ± 0.032	0.872 ± 0.016	0.852 ± 0.046	0.833 ± 0.053	**0.867** **±** **0.023**
Put.R	0.887 ± 0.023	**0.889** **±** **0.025**	0.876 ± 0.060	0.897 ± 0.008	**0.899** **±** **0.013**	0.875 ± 0.011	0.842 ± 0.056	0.825 ± 0.064	**0.883** **±** **0.009**
Pal.L	0.629 ± 0.083	0.785 ± 0.039*	**0.815** **±** **0.060**	0.671 ± 0.048	0.737 ± 0.012	**0.827** **±** **0.034**	0.557 ± 0.189	0.565 ± 0.182	**0.802** **±** **0.031**
Pal.R	0.654 ± 0.058	0.768 ± 0.055*	**0.799** **±** **0.088**	0.732 ± 0.053	0.785 ± 0.024	**0.808** **±** **0.055**	0.574 ± 0.174	0.586 ± 0.175	**0.809** **±** **0.028**
Hip.L	0.800 ± 0.025	**0.814** **±** 0.029*	0.809 ± 0.014	0.804 ± 0.044	**0.813** **±** **0.045**	0.811 ± 0.036	0.783 ± 0.037	0.797 ± 0.039	**0.804** **±** **0.015**
Hip.R	0.832 ± 0.019	**0.839** **±** 0.022*	0.810 ± 0.022	0.817 ± 0.049	**0.828** **±** **0.053**	0.826 ± 0.034	0.795 ± 0.032	0.809 ± 0.031	**0.812** **±** **0.014**
Amy.L	0.672 ± 0.041	0.685 ± 0.047	**0.721** **±** **0.054**	0.630 ± 0.041	0.686 ± 0.053	**0.736** **±** **0.090**	0.540 ± 0.130	0.601 ± 0.103*	**0.745** **±** **0.050**
Amy.R	0.644 ± 0.056	0.671 ± 0.053*	**0.707** **±** **0.052**	0.609 ± 0.074	0.637 ± 0.090	**0.756** **±** **0.08**	0.455 ± 0.097	0.520 ± 0.088*	**0.758** **±** **0.055**
Acc.L	0.695 ± 0.053	**0.707** **±** **0.060**	0.699 ± 0.081	0.694 ± 0.050	**0.744** **±** **0.036**	0.742 ± 0.069	0.646 ± 0.089	**0.658** **±** **0.084**	0.655 ± 0.099
Acc.R	0.697 ± 0.067	**0.709** **±** **0.070**	0.678 ± 0.089	0.634 ± 0.036	0.676 ± 0.042	**0.725** **±** **0.063**	0.582 ± 0.081	0.595 ± 0.073	**0.691** **±** **0.082**
Avg.	0.680 ± 0.038	**0.799** **±** 0.087*	0.799 ± 0.094	0.769 ± 0.107	0.800 ± 0.094*	**0.818** **±** **0.073**	0.688 ± 0.159	0.707 ± 0.147*	**0.802** **±** **0.083**

Structure acronyms are: Tha.L, left thalamus; Tha.R, right thalamus; Cau.L, left caudate; Cau.R, right caudate; Put.L, left putamen; Put.R, right putamen; Pal.L, left pallidum; Pal.R, right pallidum; Hip.L, left hippocampus; Hip.R, right hippocampus; Amy.L, left amygdala; Amy.R, right amygdala; Acc.L, left accumbens; Acc.R, right accumbens; Avg., average value. Significant improvements after domain adaptation over baseline are indicated with “*” and maximum DSC values are shown in bold.

Significant improvement from 0.769 to 0.800 in overall DSC was achieved using the domain adaptation to the MICCAI 2012 baseline (*p* = 2.1 × 10^−13^), when tested on the IBSR-SIEMENS dataset. Also, improvements for most of the structures were observed compared to the baseline, however, not significant (*p*>0.0036). The average DSC for FIRST was better compared to our method (*p* = 0.008), however, our domain adaptation method showed better or similar results for all structures, except for the pallidum and amygdala.

The second subset of the IBSR dataset (IBSR-GE) showed to be the most difficult to obtain better segmentation results as can be also seen in Figure 4, where the increase in DSC was smaller compared to other targets. However, significant improvements were achieved by using domain adaptation, improving the average DSC of the baseline from 0.688 to 0.707 (*p* = 6.8 × 10^−10^). Also, performance improvements were achieved for most of the structures and significant increases were observed for left thalamus (*p* = 0.002), right thalamus (*p* = 0.0009), left caudate (*p* = 0.0005), left amygdala (*p* = 0.0009), and right amygdala structures (*p* = 0.0004). The average DSC of FIRST (0.802) was significantly higher than our approach (*p* = 1.7 × 10^−15^) and similar behaviour was observed for most of the structures. Similar outcome with this sub-group of the IBSR dataset has also been noticed in Kushibar et al. (2019) which will be further discussed in section 5.

**Figure 5:**
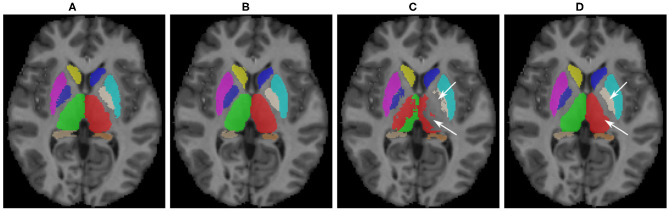
Qualitative results for sub-cortical structure segmentation: **(A)** Ground truth; **(B)** FIRST segmentation; **(C)** Pre-trained baseline CNN output without domain adaptation; **(D)** After domain adaptation. Arrows indicate: top → pallidum; bottom → thalamus.

Some qualitative results are shown in Figure 5 for the MICCAI 2012 dataset image as target. As can be seen, the baseline model did not produce satisfactory segmentation results for the thalamus and pallidum structures (indicated with arrows), which were improved after the domain adaptation. The proposed transductive domain adaptation method for segmentation greatly improved the model's performance and alleviated the segmentation errors caused by the domain-shift.

The training time for this task was 11 min on average per epoch. Additionally, the segmentation time using our method was 1.3 min (run on GPU) + 3.7 min (atlas registration, run on CPU) per volume on average. In contrast, FIRST took 10 min on average to segment all the sub-cortical structures in one subject volume.

We also tested the proposed method with the well-known U-Net architecture (Ronneberger et al., 2015) by applying the histogram loss in the features of the bottleneck layer. The average DSC for MICCAI 2012 dataset for baseline and after domain adaptation was 0.815±0.097 and 0.816±0.087, respectively. Similarly, the SIEMENS subset of the IBSR dataset yielded a DSC of 0.791±0.103 and 0.790±0.110 for baseline and TDA, respectively. A slight improvement was observed in DSC for the GE subset increasing the average from 0.738±0.129 to 0.756±0.115. A more detailed analysis will be discussed in section 5.

### 4.2. WMH Lesion Segmentation

**Figure 6:**
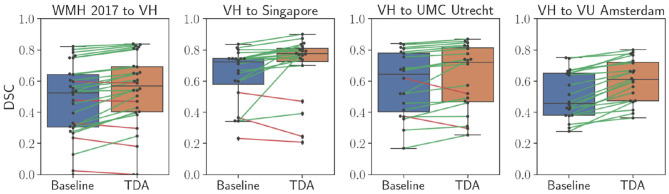
Comparison of WMH lesion segmentation between direct testing (Baseline) and after domain adaptation (TDA). Black dots refer to each subject volume in the target dataset. The connecting lines show correspondence for improved (green) and decreased (red) DSC values.

Table 2 shows quantitative results for the WMH lesion segmentation using the pre-trained baseline model without domain adaptation, our proposed domain adaptation method, and the unsupervised method LST. Additionally, Figure 6 illustrates segmentation improvements with subject-wise correspondence between baseline and domain adaptation methods for the subject volumes of the target dataset.

**Table 2:** WMH lesion segmentation results for the pre-trained baseline model without domain adaptation, transductive domain adaptation (TDA), and unsupervised LST method for four different sites: (1) source WMH 2017 and VH target; (2) source VH to Singapore; (3) source VH to UMC Utrecht; and (4) source VH to VU Amsterdam.

**WMH 2017 to VH (3T Siemens TrioTim)**				**VH to Singapore (3T Siemens TrioTim)**			
	**Baseline**	**TDA**	**LST**		**Baseline**	**TDA**	**LST**
DSC	0.478 ± 0.229	**0.536** **±** 0.232*	0.410 ± 0.232	DSC	0.636 ± 0.176	**0.703** **±** 0.198*	0.651 ± 0.176
TPR	0.735 ± 0.208	0.544 ± 0.231	0.319 ± 0.210	TPR	0.314 ± 0.089	0.451 ± 0.106	0.148 ± 0.092
FPR	0.611 ± 0.226	0.480 ± 0.256	0.477 ± 0.273	FPR	0.211 ± 0.186	0.469 ± 0.197	0.510 ± 0.153
F-score	0.270 ± 0.186	**0.308** **±** 0.187*	0.160 ± 0.140	F-score	0.265 ± 0.102	**0.289** **±** **0.118**	0.106 ± 0.067
**VH to UMC Utrecht (3T Philips Achieva)**				**VH to VU Amsterdam (3T GE Signa)**			
	**Baseline**	**TDA**	**LST**		**Baseline**	**TDA**	**LST**
DSC	0.587 ± 0.203	**0.624** **±** 0.210*	0.620 ± 0.201	DSC	0.504 ± 0.148	**0.602** **±** 0.135*	0.581 ± 0.155
TPR	0.464 ± 0.107	0.464 ± 0.148	0.250 ± 0.130	TPR	0.478 ± 0.114	0.483 ± 0.106	0.290 ± 0.105
FPR	0.279 ± 0.151	0.319 ± 0.175	0.352 ± 0.221	FPR	0.284 ± 0.155	0.298 ± 0.184	0.358 ± 0.161
F-score	0.316 ± 0.103	**0.318** **±** **0.111**	0.181 ± 0.091	F-score	0.300 ± 0.108	**0.341** **±** 0.126*	0.213 ± 0.095

DSC, dice similarity coefficient; TPR, true positive rate; FPR, false positive rate. Highest DSC and F-scores are shown in bold. Statistically significant improvements from baseline are indicated with “*”.

When the WMH 2017 dataset was used as source and VH as target, a significant improvement was achieved in segmentation, increasing the DSC from 0.410 to 0.536 (*p* = 0.0002). The F-score was significantly improved from 0.270 to 0.308 (*p* = 0.007) as was the FPR, significantly improving from 0.611 to 0.480 (*p* = 2.9 × 10^−5^), however, there was a decrease in TPR from 0.735 to 0.544 due to inter-rater variability, which will be further discussed in detail (section 5). In comparison to the DSC result for LST (0.410) and the F-score of 0.160, our method yielded significantly higher DSC (*p* = 0.001) and detection rates (*p* = 2.4 × 10^−5^) at similar operating points.

Significant improvements were obtained in lesion segmentation after applying domain adaptation from the pre-trained baseline without domain adaptation to the Singapore site, increasing the DSC from 0.636 to 0.703 (*p* = 0.006). A slight improvement was achieved in F-score but not statistically significant (*p* = 0.156). In comparison to LST, our method was significantly better in both segmentation and detection, (*p* = 0.006) and (*p* = 0.0002), respectively.

Performing domain adaptation from source VH to the target UMC Utrecht site significantly improved the DSC from 0.587 of baseline to 0.624 (*p* = 0.008). There were no improvements in lesion detection rates, and the differences in F-scores for the baseline and domain adaptation were not statistically significant (*p* = 0.794). The DSC using our method was similar to that of LST (0.620), and differences were not significant (*p* = 0.79), but significantly higher lesion detection rate was observed after domain adaptation in comparison to LST (*p* = 0.0003).

When the VU Amsterdam site was used as target, our approach achieved a significant increase in DSC, improving the baseline from 0.504 to 0.602 (*p* = 8.9 × 10^−5^). The F-score of our method with 0.341 was also significantly higher than both LST (*p* = 0.0006) and baseline (*p* = 0.0002) values, with 0.213 and 0.300, respectively. The segmentation performance of our method was slightly better than LST but not statistically significant (*p* = 0.433).

**Figure 7:**
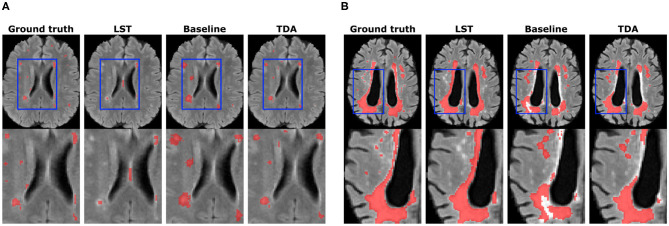
Qualitative results for WMH lesion segmentation. Small lesion **(A)** and large lesion **(B)** segmentation improvements are shown. The bottom row depicts zoomed regions of interests shown in blue rectangles on whole-brain images (top row).

Figure 7 illustrates WMH lesion segmentation examples for the pre-trained baseline without domain adaptation, after transductive domain adaptation, and unsupervised LST. As can be seen, our method produced more refined segmentation than the baseline and better detection of smaller lesions. On the other hand, LST produced more false negatives and false positives for the smaller lesions. Some false negatives for the small lesions could not be avoided even after applying domain adaptation.

In comparison to the sub-cortical structure segmentation, the number of voxels in training was varying depending on the lesion load in the source image. Since the ground truth labels are available for the source images, we handpicked a representative image with a large lesion load. It took 14 min on average per training epoch. Furthermore, the segmentation time per volume using our method was 4 min (run on GPU) + 3 min (atlas registration, run on CPU) on average. Whereas LST took 25 min on average to segment the WMH lesions in one subject volume.

## 5. Discussion

In this paper, we have introduced a novel domain adaptation method which minimises the differences in activation maps between the source and target domains in a transductive manner. As shown in Figure 2, the convolutional layers of the CNN produce different intensity distributions due to the variations in MRI images with different acquisition protocols. In order to alleviate this domain-shift effect, we performed histogram matching on the activation maps for the last convolutional layers as well as the fully connected layers of the network (Figure 1).

In the transductive domain adaptation process, we consider that manual annotations are only available for the source images, hence, optimisation of the CNN for segmentation loss can be only done using the source dataset. Therefore, the histograms of the activation maps extracted from the source were matched to those of the target. Then, the histogram loss function (Equation 1) computes how far the source feature map distributions are from the ones of target. In this way, the layers of the network are trained to produce similar activation maps to the target to minimise the distribution differences between two domains and jointly training the network to classify the input patches.

As can be seen in the results for the sub-cortical structure segmentation (Table 1), the performance of the pre-trained baseline CNN without domain adaptation was low. Moreover, this could also be observed in the segmentation example for one of the MICCAI 2012 dataset images (Figure 5), where the thalamus and pallidum structures were difficult for the network to segment. This is due to the weaker contrast between the structure boundaries and the background in comparison to other sub-cortical structures. On the other hand, the baseline segmentation for the putamen structure was better even for the baseline model. Although significant improvements were observed for both left and right putamen structures when using our domain adaptation method for the MICCAI 2012 dataset, this was not the case for the IBSR-SIEMENS and IBSR-GE datasets. However, the performance of the baseline model was similar to the one for transfer learning (Kushibar et al., 2019) due to the high contrast that this structure has compared to the background, which makes it easier for the network to generalise between different protocols. Aside from the putamen structure, our method was effective in improving the performance of the CNN for all other structures and significantly improved overall average DSC from the baseline.

The performance of the network after domain adaptation was similar to that of FIRST for the MICCAI 2012 dataset and slightly lower for the IBSR-SIEMENS dataset. However, for the IBSR-GE dataset, the result of domain adaptation was lower than that of FIRST. The MRI scans of IBSR-GE have imaging artefacts and lower quality in terms of contrast and brightness, which makes this subset of the IBSR dataset the most challenging one. In fact, the result of supervised domain adaptation using transfer learning with one image (0.784) was still lower than that of FIRST, and according to Kushibar et al. (2019), it took three images to significantly outperform FIRST using transfer learning. Since FIRST is an active-shape based model it is more robust to imaging artefacts such as motion, and can produce moderate results despite the present difficulties. However, deep-learning based supervised methods (Dolz et al., 2018; Wachinger et al., 2018; Liu et al., 2020) outperform unsupervised ones if an adequate number of images are used in training.

**Figure 8:**
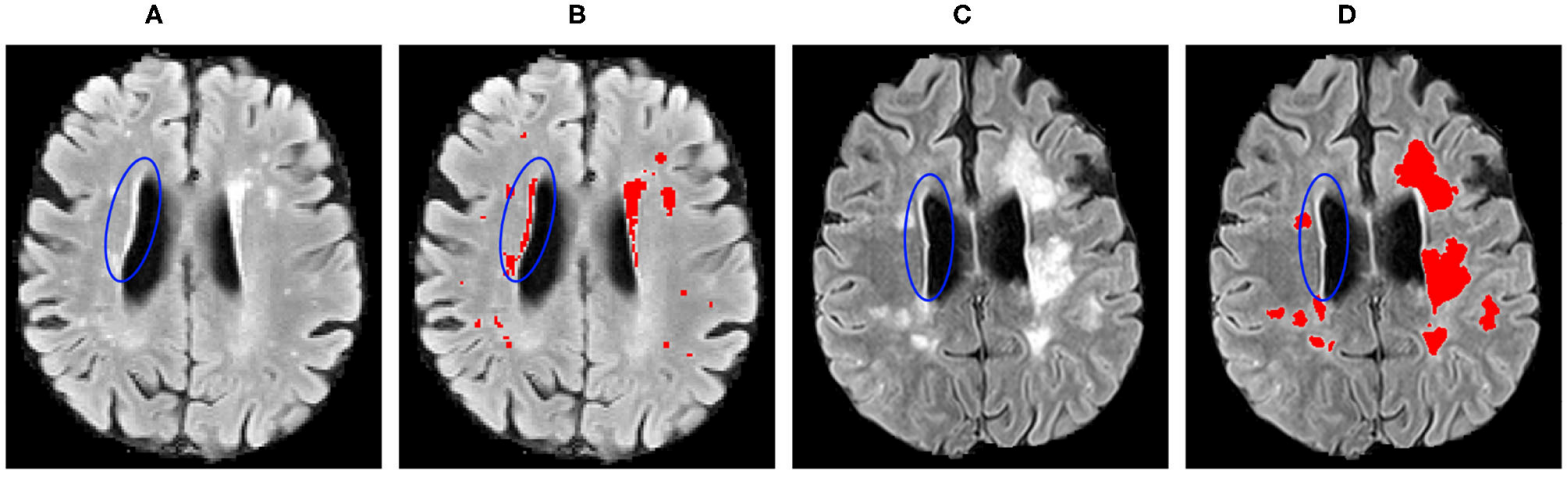
Inter-operator variability in the lesion ground truth masks for the: **(A,B)** WMH 2017; and **(C,D)** VH datasets. Blue ellipses indicate the hyperintense tissues near the ventricles.

The proposed method showed similar improvements when performing domain adaptation from pre-trained baseline model in the results for the WMH lesion segmentation task (Table 2). In general, significant improvements were observed in segmentation for all the experiments, while lesion detection was improved for some sites only. We have noticed that for this segmentation problem, inter-operator variability in the gold-standard lesion masks has an enormous effect on the lesion detection. As can be seen in Figure 8, the periventricular hyperintensities are annotated as lesions for the WMH 2017 dataset and not in VH. Moreover, there are more smaller lesions in the WMH 2017 dataset compared to the VH that have images with predominantly larger lesions. These differences introduce more difficulties in terms of better generalisation for the network and require supervised intervention to mitigate the problems of inter-operator differences between datasets.

Apart from these challenges, as shown in Figure 7, the proposed domain adaptation method significantly improved the segmentation result and produced better delineations of the lesion boundaries. Also, some smaller lesions were detected better after the domain adaptation, but some false positives still could not be avoided.

As shown in Table 2, adapting the network from WMH 2017 to the VH dataset significantly improved overall segmentation and detection rates. Also, for the images of the VH site, the results for both the baseline and domain adaptation were better than that of LST in terms of segmentation and lesion detection. However, for all the other target sites, we observed that the pre-trained baseline model without domain adaptation performed worse than LST and considerable improvements were achieved after applying domain adaptation. Overall, when adapting the model from VH to the different sites of the WMH 2017 datasets, lesion detection was not improved substantially. This was due to the inter-operator differences in the ground truths, where the CNN model was specifically trained to classify the small and periventricular hyperintense tissues as the background. However, as could be seen in Figure 6, segmentation performance was increasing for most of the subjects after applying the domain adaptation. We observed no improvement or decline in DSC for some subjects when the performance of the baseline was also low. Additionally, we observed that having at least the same scanner makes the network to be less affected by the domain shift. This could be seen in the example of NUHS Singapore site, which shares the same scanner as VH, but with different voxel resolution.

In terms of the number of images, our experiments showed that using only one image was enough for domain adaptation. This is because the histogram loss is computed only over the image features and the number of overall samples was adequate for the network to converge for both tasks. Including more training images did not improve the segmentation results due to the inter-operator variability in the expert annotated ground truths labels but increased the training time.

As could be seen in both the quantitative and qualitative results, the proposed transductive domain adaptation method is an effective way to mitigate the problems of domain-shift without the requirement for expert annotated labels. However, there are some limitations for domain adaptation when no ground truth labels are available. As we have seen in the results for the sub-cortical structure segmentation, transductive domain adaptation did not improve the DSC for structures where the performance of the pre-trained baseline model was already satisfactory to a certain degree. Similar behaviour was also observed when applying the proposed method with a commonly used U-Net architecture where the results were similar to the baseline for the MICCAI 2012 and IBSR-SIEMENS datasets. However, there was a slight improvement in the case of the IBSR-GE dataset where the baseline was affected by domain shift compared to the other sets. In general, we have noticed that U-Net was less affected by domain shift compared to our selected CNN. Moreover, it could be that the encoder-decoder architecture makes it difficult to perform TDA at the feature-level. However, the overall performance of U-Net when trained from scratch was lower than that of the 2.5D approach that achieves the state-of-the-art results for the sub-cortical structure segmentation (Avg DSC 0.85 vs. 0.87, for UNet and our method in MICCAI 2012 dataset, respectively). Further investigation on improving the feature-level domain adaptation in encoder-decoder architectures with our proposed transductive method will be taken as a future work.

Furthermore, the inter-operator variability between two datasets also makes it challenging to evaluate such approaches. We recommend applying the transductive approach for domain adaptation to overcome extreme performance drops caused by domain-shift, and when there are no manually annotated images available. Although manually annotating the MRI scans for both considered segmentation problems is a time-consuming task, supervised transfer learning approaches remain a better way to address the domain-shift problem which could be better than the traditional unsupervised methods.

In general, most of the methods in the literature address domain adaptation where the source and target images are drastically different. Moreover, there are benchmark datasets that allow such comparisons in computer vision [for example, MNIST to The Street View House Numbers (SVHN)], but we still lack such standard datasets in the medical domain. We believe some medical benchmark datasets with minimal inter-operator variability in the ground-truths masks will emerge. For example, the iSeg infant brain tissue segmentation challenge (Sun et al., 2020) and the MnM Challenge for multi-site and multi-vendor cardiac MRI segmentation (Campello and Lekadir, 2020) have recently been organised addressing this challenge. Such initiatives would definitely serve as a benchmark for domain adaptation methods. Especially for the cases when the differences in images are not drastic but still affect the performance of deep learning based methods. Also, note that in Sun et al. (2020), the reported top five methods did not propose any domain adaptation method, and the ones utilising adversarial training or CycleGAN based approaches were not among the top methods, which shows how challenging the problem is. Although these more complex methods have shown their effectiveness in multi-modality setup, there is still room for improvement in domain adaptation for multi-site single-modality cases.

## 6. Conclusions

In this paper, we have introduced a transductive transfer learning method for reducing the domain-shift effect in deep learning caused by differences in MRI scanners and image-acquisition parameters. In our approach, we computed the histogram loss defined by the differences in the histogram distributions of the activation maps for the source and target domains from the convolutional and fully connected layers of the network. Minimising the histogram loss forces the convolutional layers to produce outputs for the source which are similar to those of the target. The network is end-to-end trainable and does not require exhaustive hyper-parameter tuning.

In order to implement our pipeline, we used a network architecture recently proposed in Kushibar et al. (2018), which had shown state-of-the-art performance in sub-cortical brain structure segmentation. We employed this architecture to perform domain adaptation for two different segmentation problems. The proposed approach was tested with different experimental setups using inter-site and inter-scanner datasets.

The experimental results confirmed the effectiveness of our domain adaptation approach for two different segmentation problems, where it was possible to significantly improve the performances of the pre-trained baseline models. Performing similarly to state-of-the-art traditional unsupervised methods, our approach was able to overcome extreme performance drops caused by domain-shift problem and achieve faster segmentation process. Moreover, along with the domain-shift issue, there are differences in the manual segmentation masks, which makes evaluation of domain adaptation pipelines more challenging.

In summary, the approach presented in this work, can help to improve brain biomarker extraction for various neurological and neurodegenerative disorders, especially in clinical scenarios where manual annotation are not available. Additionally, we have made our transductive transfer learning domain adaptation pipeline available to the research community at https://github.com/NIC-VICOROB/sub-cortical_segmentation.

## Data Availability Statement

Publicly available datasets were analysed in this study. This data can be found at: https://www.oasis-brains.org/#data; https://www.nitrc.org/projects/ibsr; https://wmh.isi.uu.nl.

## Ethics Statement

The studies involving human participants were reviewed and approved by Alex Rovira, Magnetic Resonance Unit, Department of Radiology, Vall d'Hebron University Hospital, Spain. Written informed consent for participation was not required for this study in accordance with the national legislation and the institutional requirements.

## Author Contributions

KK: methodology, experiments, and writing. MS, SV, and JS: validation and review. ÀR: data provision and validation. AO and XL: supervision and review. All authors contributed to the article and approved the submitted version.

## Conflict of Interest

ÀR serves on scientific advisory boards for Novartis, Sanofi-Genzyme, Icometrix, SyntheticMR, and OLEA Medical, and has received speaker honoraria from Bayer, Sanofi-Genzyme, Bracco, Merck-Serono, Teva Pharmaceutical Industries Ltd, Novartis, Roche, and Biogen Idec. The remaining authors declare that the research was conducted in the absence of any commercial or financial relationships that could be construed as a potential conflict of interest.

## Footnotes

^1^https://www.nitrc.org/projects/ibsr.

^2^https://wmh.isi.uu.nl.

^3^http://cmictig.cs.ucl.ac.uk/wiki/index.php/NiftyReg.

^4^http://www.fmrib.ox.ac.uk/fsl.

^5^https://www.tensorflow.org.
